# Neural correlates of negative aesthetic evaluations in visual art: a neuroimaging meta-analysis

**DOI:** 10.1093/cercor/bhaf156

**Published:** 2025-07-08

**Authors:** Ryan Joseph Slaby, Maria Arioli, Marco Tettamanti, Zaira Cattaneo

**Affiliations:** Department of General Psychology, University of Padova, Via Venezia, 8, 35131, Padova, Italy; Department of Psychology, University of Milano-Bicocca, Piazza dell'Ateneo Nuovo, 1, 20126, Milan, Italy; Department of Human and Social Sciences, University of Bergamo, Piazzale S. Agostino, 2, 24129, Bergamo, Italy; Department of Psychology, University of Milano-Bicocca, Piazza dell'Ateneo Nuovo, 1, 20126, Milan, Italy; Department of Human and Social Sciences, University of Bergamo, Piazzale S. Agostino, 2, 24129, Bergamo, Italy

**Keywords:** aesthetic evaluation, aesthetic experience, displeasure, IAPS, negative affect

## Abstract

Neuroaesthetics has focused on investigating positive aesthetic evaluations while neglecting negative aesthetic evaluations. The employment of domain-general neural systems may engender hedonic valuation across an affective space of (dis)pleasure towards artistic and non-artistic stimuli. Hence, we conducted a meta-analysis assessing neural correlates associated with negative aesthetic evaluations towards visual artwork (NAE) and with viewing or evaluating negative non-artistic images from the International Affective Picture System (N-IAPS). Literature search screenings found 16 studies and 16 experiments for the NAE and 46 studies and 47 experiments for the N-IAPS. GingerALE software employed activation likelihood estimation analyses to specify neural correlates within and between NAE and N-IAPS. Meta-analytic results from the NAE solely revealed the right fusiform gyrus encroaching the anterior cerebellum, while activations across frontal, occipital, temporal and subcortical areas were revealed for the N-IAPS. A commonality between the NAE and N-IAPS was revealed within the right fusiform gyrus. These results suggest that the domain-general neural systems are at play across negative visual affective experiences, yet the context of stimulus engagement, such as aesthetic, may modulate how these neural systems are employed. Given the scarcity of results, future research in neuroaesthetics must expand from positive aesthetic evaluations to ascertain neural correlates within negative aesthetic evaluations.

## Introduction

Neuroscientific models explaining the visual aesthetic experience (henceforth aesthetic experience) generally propose that an interplay between neural systems behind sensation, reward, emotion, and cognition engenders top-down and bottom-up processes to entrain aesthetic engagement (Chatterjee and Vartanian 2014; Pelowski et al. 2016, 2017; Nadal and Skov 2025; but also see Ciricugno et al. 2023); hence, an employment of domain general systems may be functionally recruited to engage with visual artwork (Nadal and Skov 2025). These views have been supported by a plethora of meta-analyses of neuroimaging data regarding aesthetic experience (e.g. Brown et al. 2011; Vartanian and Skov 2014; Boccia et al. 2016; Chuan-Peng et al. 2020; Feng et al. 2021a; Sacheli et al. 2022; Lou et al. 2025; Vartanian et al. 2025). Critically, the constructs investigated by these meta-analyses, and likewise by neuroaesthetic literature, are largely within the domain of positivity (i.e. positive aesthetic experience), encompassing beauty, pleasure, and aesthetic appreciation. Although right within their investigation of positivity, researchers have generally neglected *negative* aesthetic experience, referring to negative aesthetic evaluations, such as ugliness, displeasure, or dislike. This negligence may be related to the general affiliation of aesthetics with the concept of beauty, hence the substantial meta-analytical evidence on the neural correlates of positive aesthetic experience.

To fill this gap, we aimed for the first meta-analysis on the neural processes associated with negative aesthetic evaluations in visual art (i.e. negative responses to questions such as: *Do you like this painting*? or *Do you find the painting beautiful*?). Importantly, negative aesthetic evaluations may be elicited by the negative affective content of artwork (e.g. slaughter of an animal) yet also by other characteristics of artwork (e.g. bad formal execution, not-preferred style, etc.), irrespective of the content. Indeed, negative aesthetic evaluations have been positively associated to the degree of an artwork’s negative affective content (Klebl et al. 2021; Dorado et al. 2023; Fekete et al. 2023), yet the subjectivity of an individual’s evaluation may oppose the negative content of a visual artwork, such as finding painful artworks beautiful (see supplementary materials in Ardizzi et al. 2021). A viewer’s employment of psychological distance during art engagement may circumvent the insurgence of intense negative affect within themselves and facilitate pleasure (Mazzocut-Mis, 2021; Menninghaus et al. 2017; see Sachs et al. 2015 for a similar argument in music). Therefore, we solely aimed to reveal neural activations distinctly associated with negative aesthetic evaluations notwithstanding the affective content depicted by visual artwork.

Negative aesthetic experience may arise from an employment of neural systems that work in concert to generate (dis)pleasure (Berridge and Kringelbach 2015; Ciricugno et al. 2023) within an affective space (Rolls 2019; Rolls et al. 2020, 2022). Indeed, pleasure and displeasure may represent poles of affective space (Barrett et al. 2007) served to compute the hedonic value derived from the sensation of looking at a painting (Skov and Nadal 2021; Ureña and Nadal 2023; Nadal and Skov 2025). The sensory valuation account for aesthetic experience surmises that three neural domain general systems are engaged for the evaluation of artwork across a displeasure-pleasure continuum (Nadal and Skov 2025). Accordingly, a sensory perception system projects visual information of an artwork to associative cortical areas that integrate an artwork’s visual information across various modalities, including physiological regulation, executive functioning and semantics. In hand, the evaluation system hedonically codes an artwork’s content in relation to the viewer’s current affective state via a broad employment of neural networks, such as the mesolimbocortical reward network, that generate (dis)pleasure across affective dimensions. A behavioral motivational system ultimately converges input from these neural systems to induce decisions and behaviors in relation to the visual stimulus in question, including aesthetic evaluations towards visual artwork (Nadal and Skov 2025). Past studies have shown an employment of the evaluation system within negative aesthetic evaluations (e.g. Kross et al. 2007; Ishizu and Zeki 2011, 2017; Osaka et al. 2012; Vessel et al. 2012; Flexas et al. 2014; Cattaneo et al. 2014a, 2014b; Vartanian et al. 2015); therefore, the evaluation system may engender negative aesthetic evaluations that are comprised by an overlay of displeasure (Nadal and Skov 2025).

Given the scant neuroimaging literature on the individual constructs of negative aesthetic evaluations, such as ugliness and disliking, we further maintained a focus on the construct of negative aesthetic evaluations as a whole to tackle the neural intricacies of displeasure within aesthetic experience. Notwithstanding the importance of considering neural activations associated to positive aesthetic evaluations elicited by artworks depicting negative affective content, here we focused our analysis on studies that reported neural activations associated with negative aesthetic evaluations (from here onward NAE), irrespective of whether these were elicited by the depicted content or by other features of the artwork. As such, studies that either assessed negative aesthetic evaluations towards art within the MRI scanner or additionally assessed the same art images by the same participants for negative aesthetic evaluations outside the scanner were included in the meta-analysis.

Considering the plethora of domain-general networks at play, we expected recruitment of the various systems implicated in the negative evaluation of artistic stimuli, particularly those comprising the evaluative system as proposed by Nadal and Skov (2025; see above). Moreover, we compared neural activations mediating negative aesthetic evaluations to neural activations associated with viewing non-artistic images of negative affect taken from the International Affective Picture System (IAPS; Lang et al. 2005). Negative IAPS (from here onward N-IAPS) images have been demonstrated to sufficiently induce negative affect within the viewer alongside a recruitment of neural systems involved in hedonic evaluation and negative emotion (Chang et al. 2015; Mansueto et al. 2025). Negative affect represents the core dimension within the negative subgroup of the image set (Lang et al. 2005; Chang et al. 2015) yet also represents the negative pole of (dis)pleasure in core affect theory (Barrett et al. 2007). As displeasure grounds negative aesthetic evaluations (Nadal and Skov 2025), we expected that neural activations in response to the negative subset of IAPS images would be akin to those elicited by negative aesthetic evaluations. Yet, given the aesthetic context of artistic stimuli, differences between the two may emerge within neural systems that perceptually code the stimulus as an artwork alongside those that compute displeasure.

## Materials and methods

### Rationale of meta-analytic approach

By utilizing the activation likelihood estimation (ALE) meta-analytical approach (Muller et al. 2018), we investigated the neural basis of the NAE and N-IAPS. The methodology of individual neuroimaging experiments can be affected by researcher bias and paradigm limitations (Carp 2012; Radua and Mataix-Cols 2012); therefore, meta-analyses offer an overarching insight into the neural mechanisms involved at large across a plethora of studies (Turkeltaub et al. 2002). Accordingly, the ALE’s statistical approach combines and analyzes coordinates from published neuroimaging studies. We conducted two individual meta-analyses: one focused on NAE and one focused on N-IAPS. We further conducted a comparison at the meta-analytic level between the results of these two individual meta-analyses. The inclusion and exclusion criteria were defined in a meeting by all authors before continuing with the literature search and study selection. The literature search and study selection were made by two independent evaluators (authors RJS and MA) and finally approved by all other authors.

### Literature search and study selection

**Fig. 1:**
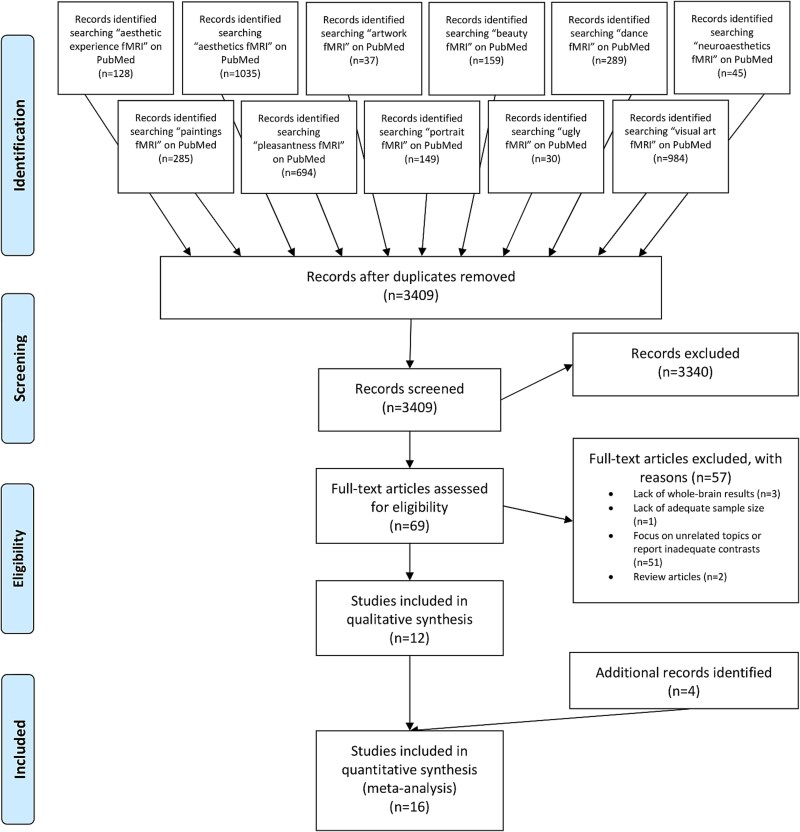
PRISMA flowchart (Moher et al. 2009) representing an overview of the study selection process for the meta-analysis on the negative aesthetic evaluation of visual artwork (NAE). Additional records identified by contacting authors from the initial study pool and through screening studies referenced by the included studies alongside review articles and meta-analyses on aesthetic experience.

**Fig. 2:**
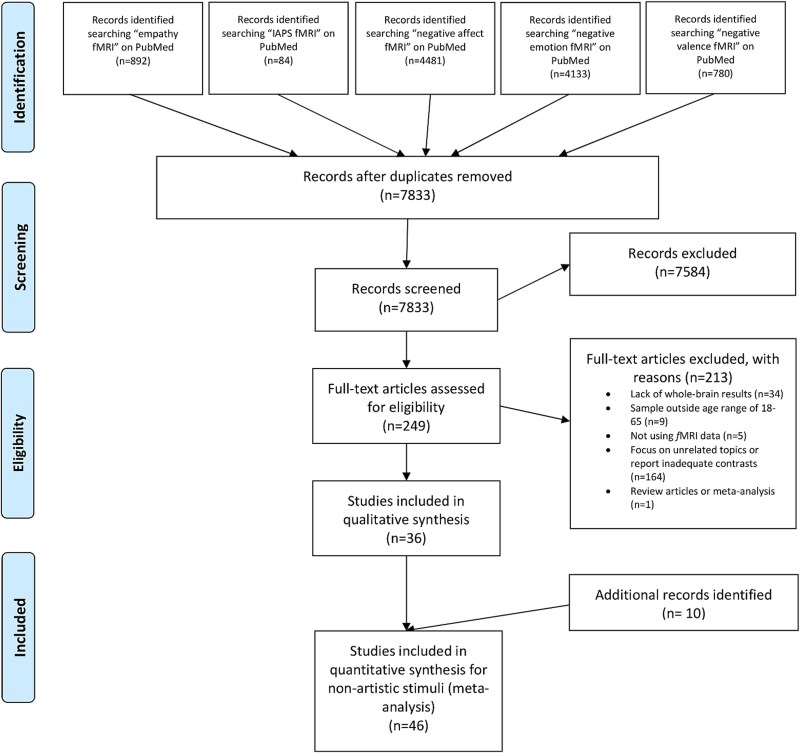
PRISMA flowchart (Moher et al. 2009) representing an overview of the study selection process for the meta-analysis on the negative international affective picture system (N-IAPS). Additional records identified by contacting authors from the initial study pool and through screening studies referenced by the included studies.

The selection of literature was initiated by using keyword strings on PubMed (https://www.ncbi.nlm.nih.gov/pubmed/; date: June 1st, 2022; see Figs. 1 and 2 for an overview of study selection). For the meta-analysis on NAE, we searched for studies by using the following keyword strings: “aesthetic experience fMRI”, “aesthetics fMRI”, “artwork fMRI”, “beauty fMRI”, “dance fMRI”, “neuroaesthetics fMRI”, “paintings fMRI”, “pleasantness fMRI”, “portrait fMRI”, “ugly fMRI”, and “visual art fMRI”. This search was further updated on March 25th, 2025 using the same exact search string. For the meta-analysis on N-IAPS, we searched for studies by using the following keyword strings: “Empathy fMRI”, “IAPS fMRI”, “negative affect fMRI”, “negative emotion fMRI”, and “negative valence fMRI”. After duplicate removal, the search strings revealed 3409 studies for the NAE and 7833 studies for the N-IAPS; these studies were further assessed for inclusion based on the following inclusion criteria:

1. Studies published in English.
2. Empirical studies using functional magnetic resonance imaging (fMRI) and a sample size of at least seven participants (Tahmasian et al. 2019).
3. Healthy and non-clinical adult populations between the ages of 18 and 65.
4. Studies reporting Montreal Neurological Institute (MNI) or Talairach coordinates from whole-brain analyses, while excluding studies that reported only analyses restricted to region of interest (ROIs) or small volume correction (SVC) analyses, as these can skew ALE results towards an unrepresentative fashion (Muller et al. 2018).
5. Studies using visual stimuli only.

Specific to the NAE, we selected studies investigating visual art-based stimuli, such as images of paintings, sculptures, drawings, photographs, pictographs, buildings, and videos of dance without audio, were included.

Specific to the N-IAPS, only studies using the IAPS were included. We decided to select only studies using the IAPS to have a homogeneous group of non-artistic studies using a well-known and validated database typically used to evoke negative affect with non-artistic stimuli (Lang et al. 2005), while we excluded other studies using different kinds of visual stimuli (e.g. ad hoc dataset as in Arioli et al. 2021a).

1. Studies using visual processing tasks.

For the N-IAPS, we selected studies using tasks visual in nature (e.g. visual discrimination, passive viewing, ratings of emotional intensity). Experiments from studies that reported results from non-visual or visual tasks outside perceptual or emotional evaluations, such as attentional (e.g. Stroop) or memory-based tasks (i.e. hits vs. misses), were excluded. For the NAE, we selected only studies in which an explicit aesthetic evaluation of artistic stimuli was required of participants that took part in fMRI experiments (either during the fMRI or outside the scanner). Also, studies investigating aesthetic evaluations of non-artistic visual stimuli (e.g., faces) were excluded, because it was not possible to clearly classify them in either the artistic or non-artistic domain (see Limitations for further discussion on this point).

1. Studies using specific contrasts.

Specific to the NAE, contrasts assessed negative versus positive aesthetic evaluations related to the subjective aesthetic evaluations provided by the participants. Specific to the N-IAPS, contrasts assessed negative versus neutral/positive affect of the IAPS images.

For the NAE meta-analysis (Fig. 1), we excluded 3340 studies from screening the titles and abstracts. Inspection of the full text, including supplementary materials, of the remaining 69 articles lead to the further exclusion of studies that lacked whole brain results (*n* = 3), had a lack of adequate sample size (*n* = 1), focused on unrelated topics/reported inadequate contrasts (*n* = 51), or were review articles (*n* = 2). 12 studies in total fulfilled the above specified criteria. After contacting six authors for unreported results, one study was further included into the study pool. Moreover, we ensured the inclusion of compatible studies that may have fell outside our initial literature search by screening studies referenced by the included studies alongside review articles and meta-analyses focused on aesthetic experience (Brown et al. 2011; Vartanian and Skov 2014; Boccia et al. 2016; Chuan-Peng et al. 2020; Feng et al. 2021a; Sacheli et al. 2022; Lou et al. 2025; Vartanian et al. 2025). Accordingly, three additional studies met our inclusion criteria and yielded our final number of included studies to 16 with an overall of 278 subjects and 98 foci (see Table 1 for an overview of the included studies).

**Table 1:** An overview of the studies included in the meta-analysis on the negative aesthetic evaluation of visual artwork (NAE).

**N**	**Author and year**	**Sample size (mean age of participants)**	**Stimuli**	**Task**	**Contrast**	**Human (H), non-human (N), or both (B) content**	**Number of Foci included in ALE**
1	Bao et al. 2017	16 (24.45)	Paintings	Passive viewing; Outside MRI: Aesthetic Appreciation	Altered Green Paintings > Original Red Paintings by Chinese Artist LaoZhu; Participants’ evaluation: altered paintings preferred less than original	H	3
2	Bermudez et al. 2017	12 (age range: 26–48)	Architectural Spaces	Passive viewing; Outside MRI: Interview and questionnaire about appreciation, relaxation, peace and beauty	Ordinary > Baseline; Ordinary (Control) > Contemplative Architecture (Experimental); Participants’ evaluation: ordinary architectural spaces described negatively (i.e. ugly), while contemplative architectural spaces described positively (i.e. beautiful)	N	16
3	Di Dio et al. 2007	14 (24.5)	Sculptures	Aesthetic Evaluation of Beauty	Ugly > Beautiful (Main effect and interaction for Aesthetic Judgment)	H	6
4	Di Dio et al. 2011	24 (28.28)	Sculptures	Aesthetic Evaluation of Beauty	Modified > Canonical Sculptures; Participants’ evaluation: modified sculptures rated as more ugly than canonical sculptures	H	2
5	Di Dio et al. 2016	19 (21.96)	Paintings	Observation (indicate when a red circle appears); Aesthetic Evaluation of Beauty; Judgment of Movement	Paintings of Humans > Paintings of Nature (Observation, Aesthetic Judgment, Movement Judgment); Participants’ evaluation: artwork of human stimuli received lower aesthetic ratings of valence than paintings of nature	B	13
6	Flexas et al. 2014	24 (23.54)	Paintings and professional photography	Aesthetic Evaluation of Beauty	Abstract > Representational; Artwork Participants’ evaluation: abstract artwork rated as not beautiful more than representational artwork	B	3
7	Ishizu and Zeki 2011	21 (27.5)	Paintings	Aesthetic Evaluation of Beauty	Visually Ugly > Visually Beautiful; Classification based on participant ratings of stimuli for validation and same participants underwent scanning session	B	7
8	Ishizu and Zeki 2017	21 (28.6)	Photography (professional photographers of national geographic)	Aesthetic Evaluation of Beauty	Sorrowful Beauty > Joyful beauty; Classification based on participant ratings of stimuli for validation and same participants underwent scanning session	B	9
9	Kawabata et al. 2004	10 (20–31)	Paintings	Aesthetic Evaluation of Beauty	Ugly > Beautiful; Classification based on participant ratings of stimuli for validation and same participants underwent scanning session	B	2
10	Kross et al. 2007	20 (24.5)	Paintings	Passive viewing	Paintings displaying rejection > Paintings displaying acceptance; Paintings validated by MRI participants for feelings of distress (collapsed variable of pleasant/secure vs. unpleasant/insecure).	H	12
11	Thakral et al. 2012	16 (18–65)	Paintings	Aesthetic Evaluation of Pleasantness	Unpleasant > Pleasant (based on participants’ evaluation)	B	8
12	Vartanian and Goel, 2004	12 (28)	Paintings	Aesthetic Evaluation of Preference	Negative correlation with preference rating	B	1
13	Vartanian et al. 2015	18 (23.39)	Architectural Spaces	Aesthetic and Approach-Avoidance Evaluations	Enclosed Spaces > Open Spaces Participants’ evaluation: enclosed spaces rated as less beautiful	N	1
14	Vessel et al. 2012	16 (27.6)	Paintings	Aesthetic Evaluation of Feeling Moved	Correlation of “Negative Emotional” PCA Factor with Whole Brain contrast of for the aesthetic evaluation of being moved > not being moved	B	2
15	Zhang et al. 2016	16 (21.29)	Chinese Pictographs	Aesthetic Evaluation of Beauty and Evaluation of Luminance	Ugly Pictograph > Low Luminance; Participants’ evaluation: low beauty for ugly pictographs and negative oracle bone scripts	N	2
16	Zhang et al. 2017	19 (21.74)	Chinese Pictographs and Oracle Bone Scripts	Aesthetic Evaluation of Beauty and Evaluation of Luminance	Ugly Pictograph > Low Luminance; Negative Oracle Bone Script > Low Luminance; Negative Oracle Bone Script > Positive Oracle Bone Script; Participants’ evaluation: low beauty for ugly pictographs; negative social meaning for negative vs. positive bone scripts	N	11
Total number of experiments: 16		Total number of participants: 278				H = 4; N = 4 B = 8	Total number of foci: 98

For the N-IAPS (Fig. 2), we excluded 7584 studies from screening the titles and abstract. Further inspection of the full text and supplementary materials of the remaining 249 articles resulted in the further exclusion of studies that lacked whole brain results (*n* = 34), lacked an adequate sample (*n* = 9), did not use fMRI data (*n* = 5), focused on unrelated topics/reported inadequate contrasts (*n* = 164), or were review articles (*n* = 1). 36 studies fulfilled the inclusion criteria. After contacting 24 authors for unreported results, three studies were further included into the study pool, and through screening studies referenced by the included studies, seven studies were further included yielding a final number of 46 included studies (resulting in 47 experiments) with 2555 subjects and 657 foci (see Table 2 for an overview of the included studies).

**Table 2:** An overview of the studies included in the meta-analysis on the negative international affective picture system (N-IAPS).

**N**	**Author and year**	**Sample size (mean age of participants)**	**Stimuli**	**Task**	**Contrast**	**Human (H), non-human (N), or both (B) content**	**Foci included in ALE**
1	Aldhafeeri et al. 2012	15 (44.5)	IAPS	Passive Viewing	Unpleasant > Baseline	B	16
2	Andreano et al. 2010	17 (20.88)	IAPS	Passive Viewing	Emotional (Negative) > Neutral	H	8
3	Barro ´s-Loscertales et al. 2010	45 (21.82)	IAPS	Passive Viewing and Letter Discrimination Task	Aversive > Neutral	B	18
4	Beraha et al. 2012	36 (36.7)	IAPS	Passive Viewing; half of trials with attentional cues for emotion	Lateralization: Negative > Neutral; Negative > Positive	B	7
5	Bisby et al. 2016	20 (23)	IAPS	Passive Viewing followed by intrascanner indication if the presented two images go well/are plausible together; second memory recognition task	Negative > Neutral (Encoding only)	H	7
6	Blair et al. 2007	22 (27.95)	IAPS and arabic symbols of numbers	Passive Viewing	Negative > Neutral + Positive	B	6
7	Bo et al. 2021	20 (20.4)	IAPS	Passive Viewing	Unpleasant > Pleasant	B	28
8	Canli et al. 1998	8 (25.6 for 14 parts; 18–65)	IAPS	Judgment of arousal and valence	Negative > Positive	B	5
9	Diers et al. 2021	46 (18–38)	IAPS, EmoPicS (emotional picture set)	Emotional Regulation (Distance or Permit)	Negative Stimulation Phase > Neutral Stimulation Phase	B	9
10	Domes et al. 2010	33 (24.89)	IAPS	Passive viewing	Negative > Neutral	B	24
11	Erk et al. 2006	14 (21–25)	IAPS	Working Memory Task followed by picture presentation	Negative > Neutral for picture presentation	B	11
12	Ferri et al. 2013	41 (22.29)	IAPS	Passive viewing then rate negative emotion	Unpleasant No Focus > Neutral No Focus (both experiments)	B	25
13	Gerdes et al. 2010	17 (25.12)	IAPS	Passive viewing then rate pleasantness post-scanning	Unpleasant > Neutral; Pleasant	B	4
14	Herwig et al. 2007	16 (23–36)	IAPS	Passive viewing after emotional cue	Expectation Negative > Neutral	B	15
15	Kark et al. 2015	17 (23.9)	IAPS	View and decide to approach or withdrawal from depicted scene; then recognition task	Negative Hits > Neutral Hits	B	19
16	Koenigsberg et al. 2010	16 (31.8)	IAPS- social scenes	Suppress or maintain, then image viewing, then rate emotional reaction in valence	Negative > Neutral	H	8
17	Kolesar et al. 2017	13 (18–30)	IAPS	Passive Viewing	Negative > Neutral	B	10
18	Kornelsen et al. 2019	20 (22.0)	IAPS	Passive Viewing and rate pain from noxious stimuli on 10th pulse	Negative > Neutral; Positive	B	12
19	Kuniecki et al. 2018	19 (22.9)	IAPS & Nencki Affective Picture System	Passive viewing with attentional check of image being outdoors or indoors	Negative > Neutral (Pupil dilation and Image Noise)	B	9
20	Lee et al. 2004	10 (29.5)	IAPS	Emotional Intensity	Negative > Neutral	B	9
21	Lee et al. 2010	13 (25–39)	IAPS	Either choose the truth or lie about picture valence	True Negative > True Positive	B	17
22	Lemogne et al. 2011	45 (23.3)	IAPS, EPS (Empathy Picture System), Images of indoors/outdoors	Viewing and Judgment (Valence to the self, Valence in general, control = indoors or outdoors)	Negative > Positive	B	4
23	Loos et al. 2019	1385 (22.38)	IAPS	Picture encoding task (passive viewing then rate valence and arousal)	Negative > Neutral (encoding)	B	16
24	Mataix-Cols et al. 2008	37 (30.7)	IAPS	Imagine self in the situation and then rate anxiety	Disgust > Neutral (positive correlations)	B	15
25	Mériau et al. 2009	23 (27.1)	IAPS	Passive viewing	Aversive > Neutral	B	8
26	Meseguer et al. 2007	14 (28.8)	IAPS	Passive view then letter discrimination task	Negative > Neutral	B	35
27	Moore et al. 2019	22 (18–31)	Shapes stimuli and IAPS distractors	Visual oddball task	Negative Distractors > Neutral Distractors	B	44
28	Nielen et al. 2009	23 (20–25)	IAPS	Stimulus classification (outdoors vs. indoors)	Negative > Positive	B	18
29	Ochsner et al. 2009	20 (20.3)	IAPS	View then rate emotion	Increased negative affect rating correlated with activation from Bottom-up Negative > Bottom-up Neutral contrast	B	2
30	Pedale et al. 2019	22 (23.6)	IAPS (No Humans)	Passive viewing (Localizer Task)	Negative > Neutral + Positive (1 experiment)	N	10
31	Radua et al. 2014	40 (19–59)	IAPS	Look/Passive viewing	Disgust > Neutral; Fear > Neutral; Sadness > Neutral	B	9
32	Reisch et al. 2020	43 (32.19)	IAPS, FACES database, words	Passive viewing, then at the end, rate random selection of 10 items for valence and arousal	Negative > Neutral: 2 analyses for IAPS and FACES	B	9
33	Schmitz et al. 2009	16 (22.03)	IAPS	Passive viewing	Negative > Positive	H	1
34	Silvers et al. 2015	30 (21.97)	IAPS	View and rate negativity	Look Negative > Look Neutral	B	32
35	Smith et al. 2004	15 (18–32)	IAPS	Recognition of previously seen (hits vs. misses)	Negative > positive (Hits)	B	8
36	Sokołowski et al. 2022	83 (21.66)	IAPS	Passive Viewing (Look condition in cognitive reappraisal task)	Negative > Neutral; Negative > Positive	B	33
37	Sterpenich et al. 2014	34 (22.15)	IAPS	Viewing then rate for pleasantness after picture disappeared	Negative > Neutral; Positive	B	23
38	Thomas et al. 2019	30 (18–65)	IAPS	Watch during scan; rate emotional valence and intensity after scan	Negative > Neutral	H	32
39	Uchida et al. 2015	62 (22.3)	IAPS	Attend and rate the negative emotion experienced	Attend Negative > Attend Neutral	B	5
40	Van Dillen et al. 2009	17 (20)	IAPS	Passive viewing then arithmetic problem and mood rating	Negative > Fixation	B	18
41	Villalta-Gil et al. 2017	32 (23.13)	IAPS	Cued Aversive Picture Task (CAP); Aversive and Erotica Picture Task (AEP)	CAP Negative > Neutral; AEP Negative vs. Neutral	B	3
42	Waugh et al. 2014	27 (23.8)	IAPS	Passive Viewing Picture, rate emotional intensity 2nd the picture (all same valence)	Negative > Neutral (Non-maintain)	B	6
43	Weierich et al. 2010	15 (22.2)	IAPS	Viewing and Arousal Rating	Negative > Neutral	B	10
44	Wittfoth et al. 2020	17 (23.47)	IAPS	Passive viewing then rate negativity with or without emotional regulation strategy	Fear & Disgust> Neutral	B	18
45	Wrase et al. 2003 (Women)	10F (40)	IAPS	Passive viewing	Negative > Neutral	B	8
46	Wrase et al. 2003 (Men)	10 M (43.2)	IAPS	Passive viewing	Negative > Neutral	B	3
47	Zhang et al. 2015	25 (18.61)	IAPS	View then rate arousal	Negative > Neutral	B	20
Total number of experiments: 47		Total number of participants: 2555				H = 5 *n* = 1 B = 41	Total number of foci: 657

A pressing methodological issue concerns the presence of multiple experiments using the same participants in one study. Therefore, all coordinates from studies using the same participants throughout multiple experiments were coded as single experiments. This resulted in the provision of 16 studies and 16 experiments for the NAE and 46 studies and 47 experiments for the N-IAPS.

### Activation likelihood estimation

GingerALE software 3.0.2 was utilized to conduct ALE analyses to specify neural correlates associated within and between the NAE and N-IAPS via the method recommended by Eickhoff et al. (2012).

In each meta-analysis, all coordinates were reported or transformed to MNI space via the provided automatic routine within in GingerALE. Afterwards, activation foci were interpreted as the central points of 3D Gaussian probability distributions, capturing the spatial uncertainty associated with each specific coordinate. These 3D probabilities representing activation foci in each experiment were combined to construct modeled activation maps (MA), which were combined to produce the ALE scores that represented the convergence across all included experiments in each brain voxel (Turkeltaub et al. 2002). ALE scores were then contrasted with an empirically defined null distribution to determine true convergence from random convergence (i.e. noise; Eickhoff et al. 2012). Accordingly, the null distribution represented the random spatial association between experiments, while the ALE scores represented the fixed distribution of foci within experiments. The application of this random-effects inference demonstrated the above-chance convergence between experiments and not on the clustering of foci within experiments. Therefore, each MA had a random voxel sampled and the union of resulting values were calculated and recorded. The derived “random” ALE scores were retained to provide a sufficient sample of the ALE null distribution. To further correct for multiple comparisons and reduce the chance of reporting false positives, the true ALE scores were tested against the random ALE scores at a conventional threshold of *P* < 0.05 corrected for cluster-level family-wise error, and at a conservative cluster-forming threshold of *P* < 0.001 with 1000 permutations (as in Arioli et al. 2022).

The resulting significance maps of ALE scores represented the individual activation maps of foci for the two meta-analyses (NAE and N-IAPS meta-analyses). These maps were further used for direct comparison and conjunction analysis to reveal the common and specific neural correlates for NAE and N-IAPS. As inputs, we entered the ALE images resulting from the separate meta-analyses NAE and N-IAPS. This is accomplished by first generating a conjunction image, representing common brain activations between NAE and N-IAPS (Eickhoff et al. 2011). In the same analysis, GingerALE randomly divided the foci of the two original datasets in two new datasets, preserving their sizes. For each of the new dataset, an ALE image was created, which was then subtracted from the other one and compared with the original data. Contrast analyses adopted a threshold of *P* < 0.001 with a minimum cluster volume size of 20m^3^ and 1000 permutations (as in Sacheli et al. 2022).

GingerALE produces anatomical labeling of all clusters alongside peak voxels and the associated statistical scores. Furthermore, Statistical Parametric Mapping Anatomy Toolbox (v.2.2c; Eickhoff et al. 2005), alongside the AAL template (as implemented in MRIcron; https://www.nitrc.org/projects/mricron) and Neurosynth (https://www.neurosynth.org/locations/), were utilized to confirm GingerALE’s localizations.

## Results

### Meta-analysis on the negative aesthetic evaluation of visual artwork

**Fig. 3:**
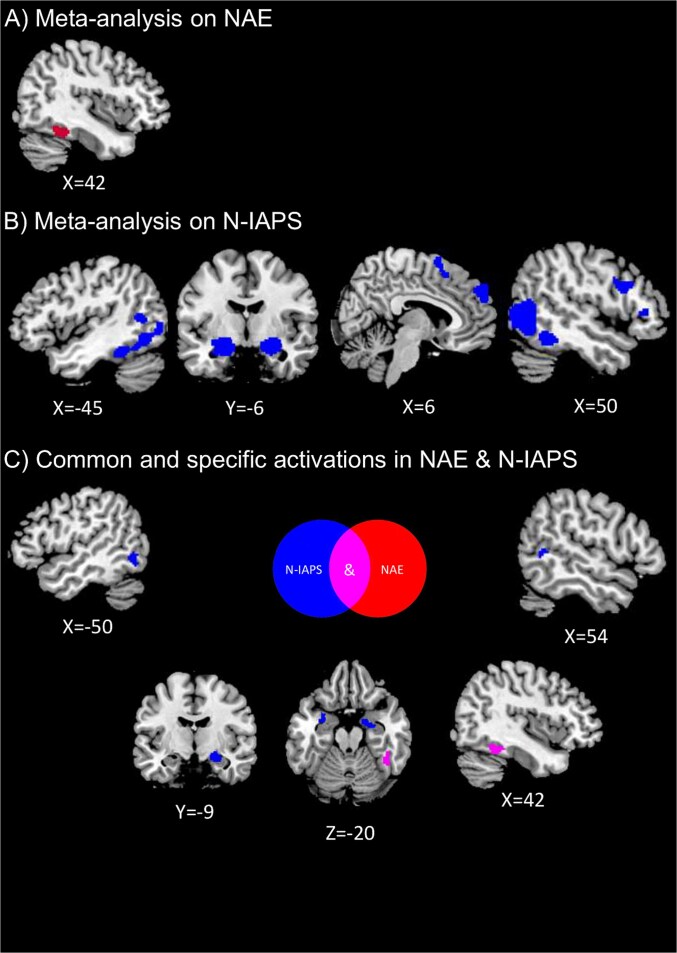
Brain regions involved in the negative aesthetic evaluations of visual art (A; NAE), the negative international affective picture system (B; N-IAPS), and the specific and common activations between the NAE and N-IAPS (C). Coordinates are reported within MNI space.

For the NAE, we solely reported consistent activations in a cluster involving the right fusiform gyrus encroaching the right anterior cerebellum (Table 3, Fig. 3A).

**Table 3:** Significant results of the meta-analysis on the negative aesthetic evaluation of visual artwork (NAE).

**Cluster #**	**Volume (mm** ^**3**^ **)**	**x**	**y**	**z**	**Brain regions**
1	744	42	−42	−22	Right Fusiform Gyrus
		42	−48	−20	Right Fusiform Gyrus
		42	−48	−24	Right Fusiform Gyrus and Right Cerebellum (Anterior Lobe)

Note: The following reported for each cluster: number, volume size (in mm^3^), MNI coordinates and anatomical labeling.

### Meta-analysis on the negative international affective picture system

The N-IAPS revealed consistent activations in the temporal areas, involving the superior, middle and inferior temporal gyri. In the right hemisphere, the meta-analytic activation extended to the right fusiform and to the right inferior occipital gyrus (Table 4, Fig. 3B). In the left hemisphere, the meta-activation involved the left fusiform gyrus and the inferior and middle temporal gyri, alongside the middle occipital areas and the anterior cerebellum (Table 4, Fig. 3B). A further activation was found in the right frontal lobe, particularly involving precentral gyrus as well as the inferior, medial and superior frontal gyri (Table 4, Fig. 3B). Finally, we found activations within the bilateral amygdala encroaching parahippocampal gyri (Table 4, Fig. 3B).

**Table 4:** Significant results of the meta-analysis on the negative international affective picture system (N-IAPS).

**Cluster #**	**Volume (mm** ^**3**^ **)**	**x**	**y**	**z**	**Brain regions**
1	8504	52	−64	2	Right Middle Temporal Gyrus
		44	−48	−20	Right Fusiform Gyrus and Right Cerebellum (Anterior Lobe)
		52	−74	2	Right Inferior Temporal Gyrus
		46	−60	−12	Right Fusiform Gyrus
		42	−58	−14	Right Fusiform Gyrus
		46	−76	−8	Right Inferior Occipital Gyrus
		56	−52	6	Right Superior Temporal Gyrus
2	5920	−48	−66	−8	Left Middle Occipital Gyrus
		−42	−44	−20	Left Fusiform Gyrus and Left Cerebellum (Anterior Lobe)
		−52	−70	4	Left Inferior Temporal Gyrus
		−50	−74	−2	Left Middle Occipital Gyrus
		−46	−60	10	Left Middle Temporal Gyrus
		−42	−56	−18	Left Fusiform Gyrus
3	4328	26	−6	−16	Right Amygdala/Parahippocampal Gyrus
4	4192	−20	−6	−16	Left Amygdala/Parahippocampal Gyrus
5	2552	42	8	28	Right Precentral Gyrus
6	1968	6	58	38	Right Medial Frontal Gyrus
		4	52	36	Right Medial Frontal Gyrus
		6	58	26	Right Superior Frontal Gyrus
7	1360	54	32	4	Right Inferior Frontal Gyrus
		54	26	10	Right Inferior Frontal Gyrus
8	1024	8	10	68	Right Superior Frontal Gyrus
		6	18	52	Right Superior Frontal Gyrus

Note.: The following reported for each cluster: number, volume size (in mm^3^), MNI coordinates and anatomical labeling.

### Conjunction and contrast analyses between the negative aesthetic evaluation of visual artwork and negative international affective picture system

We found consistent and common activations in a cluster involving the right fusiform gyrus and the right anterior cerebellum, for both the NAE and N-IAPS (Table 5, Fig. 3C). While we did not find any significant differences in comparing NAE minus N-IAPS, the reverse contrast highlighted the consistent activation of the left middle occipital cortex extending to the inferior temporal lobe, as well as in the right fusiform gyrus and in the superior temporal sulcus and, subcortically, in the bilateral amygdala encroaching parahippocampal gyri (Table 5, Fig. 3C).

**Table 5:** Common and specific regions across the meta-analyses on the negative aesthetic evaluation of visual artwork (NAE) and the negative international affective picture system (N-IAPS).

**NAE & N-IAPS**					
**Cluster #**	**Volume (mm^3^)**	**x**	**y**	**z**	**Brain regions**
1	616	42	−42	−22	Right Fusiform Gyrus
		42	−48	−20	Right Fusiform Gyrus
		42	−48	−24	Right Fusiform Gyrus and Right Cerebellum (Anterior Lobe)
**N-IAPS > NAE**					
**Cluster #**	**Volume (mm^3^)**	**x**	**y**	**z**	**Brain regions**
1	2184	21	−9	−14	Right Amygdala/Parahippocampal Gyrus
		25	−10	−15	Right Amygdala/Parahippocampal Gyrus
2	664	−49	−66	−6	Left Middle Occipital Gyrus
		−52	−70	−2	Left Inferior Temporal Gyrus
3	296	−30	5	−20	Left Amygdala/Parahippocampal Gyrus
		−29	−1	−20	Left Amygdala/Parahippocampal Gyrus
4	176	55	−54	5	Right Superior Temporal Gyrus
5	64	48	−66	−10	Right Fusiform Gyrus

Note.: The following reported for each cluster: number, volume size (in mm^3^), MNI coordinates and anatomical labeling. The contrast of NAE > N-IAPS revealed no significant clusters.

## Discussion

Given the neglect towards exploring the neural correlates of negative aesthetic evaluations towards visual art (NAE), we conducted a meta-analysis to assess the NAE alongside its neural similarities and differences towards a control counterpart of negative non-artistic stimuli distinctly comprised by the International Affective Picture System (N-IAPS; Lang et al. 2005).

### Meta-activations within the negative aesthetic evaluation of visual artwork

Regarding the NAE, our results surprisingly showed sole activation within the midst of the right fusiform gyrus. This region lies within the ventral visual pathway (Arcaro et al. 2009) and exhibits dominance in the perceptual expertise of objects, across abstract (Slotnick and White 2013) and representative categories (Gilaie-Dotan et al. 2012; McGugin et al. 2014). Study contributors of NAE’s right fusiform cluster explored the unpleasantness of Van Gogh paintings (Thakral et al. 2012), human paintings of negative valence (Di Dio et al. 2016), and ugly representative paintings (Ishizu and Zeki 2011). Authors of the contributing studies suggest that temporo-occipital areas may engender perceptual processes, such as the implicit and explicit classification of visual artwork (Ishizu and Zeki 2011; Di Dio et al. 2016; Pelowski et al. 2017). However, the temporo-occipital cortex has been implicated to represent both the salience (Bradley et al. 2012) and intrinsic stimulus properties of affective stimuli (McTeague et al. 2015). Hence, the right fusiform gyrus has been shown to be engaged during prolonged engagement with fearful (Riegal et al. 2022) and sad faces (Nara et al. 2025), perhaps via coupling between the temporo-occipital cortex and amygdala to detect and process stimuli of negative salience (Bo et al. 2021; Britton et al. 2008; Feng et al. 2021b; Pessoa and Aldophs, 2010). Therefore, the recognition of a visual stimulus as an artwork may engender patterns within the temporo-occipital cortex (see also Cattaneo et al. 2015, 2017) that propagate cascading effects to hedonically code displeasure towards visual artwork. An assignment of a visual stimulus’s appropriate hedonic value may depend on the context of the visual stimulus, such as artwork, which may be pivotal towards the type of engagement one undergoes (Skov, 2021; Ureña and Nadal 2023; Nadal and Skov 2025).

Moreover, the right anterior cerebellum was implicated within the NAE’s right fusiform cluster. The cerebellum has been posited to play an essential role in aesthetic experience through the call of internal models situated within limbic, subcortical, and associative cortical areas via cerebellar-thalamo-cortical loops (Adamaszek et al. 2022; Buckner et al. 2011; Habas et al. 2009; Palesi et al. 2020). Indeed, the cerebellum has been implicated within the aesthetic evaluation of visual artwork (Di Dio et al. 2007, 2011; Lutz et al. 2013; Mizokami et al. 2014), including joyful and sorrowful beauty (Ishizu and Zeki 2017). The anterior cerebellum facilitates sensorimotor function, such as mirroring to understand another’s actions (Habas et al. 2009; Van Overwalle et al. 2014; King et al. 2019), and further demonstrates intra-cerebellar connections with its posterior counterpart that is involved in higher cognitive, social and affective functions (Adamaszek et al. 2017; Cattaneo et al. 2022; Ciricugno et al. 2024; Ferrari et al. 2018, 2022a, 2022b; Van Overwalle, 2020). Given the cerebellum’s prominent role in detecting, feeling, and engaging with negative stimuli and their situational contexts (Seeley et al. 2007; Habas et al. 2009; Schutter et al. 2012; Habas 2018; Ferrari et al. 2023), the revealed activation of the anterior cerebellum may reflect an employment of domain-general systems (Adamaszek et al. 2022; Nadal and Skov 2025) to engage and mediate displeasure during the negative evaluation of visual artwork. It must be noted that this revealed activation may be an artifact resulting from the spread of proximal meta-activation from the right fusiform gyrus, especially as around 60% of neuroimaging studies have excluded the cerebellum from their analyses (Wang et al. 2025).

### Meta-activations within the negative international affective picture system

Our N-IAPS meta-analysis revealed neural activations across multiple regions, including bilateral activations within the amygdala, occipital and temporal gyri (including the anterior cerebellum), as well as right lateralized activations within the frontal lobe. Replicating previous meta-analyses on negative affect in images, including the IAPS (Feng et al. 2021b; Mansueto et al. 2025), these results suggest that our meta-analysis of non-artistic stimuli represents a strong control of negative affect.

In a similar vein as artistic stimuli, bilateral temporo-occipital activations may reflect synthetization of visual information across associative semantic stores to facilitate the recognition and inference of visual emotional stimuli (Bo et al. 2021; Pessoa and Aldophs, 2010). This is further corroborated by the meta-activations within the bilateral amygdala, implicating recursive feedback loops between the amygdala and the temporo-occipital cortex to code a visual stimuli’s salience and negativity (Bo et al. 2021; Feng et al. 2021b; Lang and Bradley 2010). Moreover, the revealed right lateralized frontal activations align with past research linking this activation towards negative emotional stimuli (Feng et al. 2021b; Mansueto et al. 2025). Accordingly, the right inferior frontal gyrus is associated with executive function and emotional regulation (Corbetta and Shulman 2002; Menon and Uddin 2010; Peña-Gómez et al. 2011; Shen et al. 2020) and may employ attentional and perceptive faculties to process negative stimuli (Cinq-Mars et al. 2022; Feng et al. 2021b; Fox et al. 2006). Activation within the medial frontal gyrus coincides with the dorsomedial prefrontal cortex, which has been associated with the hedonic evaluation of both negative and positive affective states (Mansueto et al. 2025), particularly towards others (Buckner et al. 2008; Molenberghs et al. 2016; Arioli et al. 2021b). Furthermore, activations within inferior, superior and precentral gyri may suggest an induction of motor resonance (Nummenmaa et al. 2008; Fiori et al. 2020; Ardizzi et al. 2021) reflecting a firsthand experience of negative emotion (Lamm et al. 2011; Ricciardi et al. 2013; Chang et al. 2015; Eisenberger 2015). These frontal activations are part of functional networks integrating subcortical structures (Buckner et al. 2008; Hofmann et al. 2012; Uddin et al. 2017); hence, domain general neural systems may allocate affective information across functional networks to hedonically code the value of a visual stimulus that induces negative affect (Feng et al. 2021a; Fiori et al. 2023; Nadal and Skov 2025; Mansueto et al. 2025).

### Similarities and differences between the NAE and N-IAPS

Of interest, the conjunction analysis of the NAE and N-IAPS revealed a similar cluster to that reported within the NAE. Therefore, a commonality in the processing of artistic and non-artistic stimuli may partly lie within negative affective functionality of the temporo-occipital cortex (Bradley et al. 2012; McTeague et al. 2015; Nara et al. 2025; Riegal et al. 2022) via its coupling with subcortical systems (Bo et al. 2021; Lang and Bradley 2010). Given the inclusion of the anterior cerebellum and its neural dynamics across sensorimotor, social, and affective functions (Adamaszek et al. 2017; Van Overwalle, 2020), domain-general neural systems may be employed across negative visual experiences (Feng et al. 2021b; Mansueto et al. 2025; Nadal and Skov 2025). Of interest, our contrast of NAE > N-IAPS revealed no significant activations, while the reverse contrast of N-IAPS > NAE revealed activations within the bilateral amygdala alongside temporal and occipital gyri. These results may suggest that visual and subcortical coupling may more strongly serve the hedonic coding of negative visual images versus displeasurable artwork. Indeed, negative images were found to induce stronger activations within the amygdala compared to words of negative affect, suggesting a stronger biological relevance in the vividness of negative affect in images (Feng et al. 2021b; Kensinger et al. 2011; Yonelinas and Ritchey 2015). Partial towards the amygdala’s evolutionary function in detecting threat for survivability (Wood et al. 2014), the distance employed during aesthetic engagement with visual artwork may cater an individual’s experience to be of a diminished negative arousal (Menninghaus et al. 2017; Mazzocut-Mis 2021).

### A need to expand neuroaesthetics: Insights from past research

In interpreting our findings, it is important to consider that the conjunction and contrast results lie within a lack of the NAE’s power: the NAE has weak power as demonstrated by its low amount of foci (98) and small sample size (278); while the N-IAPS demonstrates much stronger power with a high amount of foci (657) and large sample size (2555). Hence, we found strong support of the evaluation system (Nadal and Skov 2025) within our meta-analysis on N-IAPS, yet contrary to our expectations, we found no support of the evaluation system within the NAE. Despite this, previous literature investigating the neural correlates of negative aesthetic evaluations towards visual artwork begs to differ.

The evaluation system superimposes across various neural networks (Nadal and Skov 2025), including the mesolimbocortical reward, salience, and default mode networks. The mesolimbocortical reward network employs cold spots for pleasure, such as heightened activations within the caudate and striatum towards disgust (Sesack and Grace 2010; Berridge and Kringelbach 2015), and the striatum and caudate have been activated within the negative aesthetic evaluations towards visual artwork, including ugliness (Ishizu and Zeki 2011; but see also Vartanian et al. 2015), dislike (Vartanian and Goel, 2004), and negative emotional instances of feeling moved (Vessel et al. 2012). The salience network employs the anterior cingulate cortex (ACC), fronto-insular cortex (Berntson and Khalsa 2021), and amygdala (Dvash and Shamay-Tsoory 2014) to feel one’s own and another’s, particularly negative, emotions (Bernhardt and Singer 2012; Uddin 2015; Uddin et al. 2017; Fallon et al. 2020), while the default mode network, with main hubs in the medial prefrontal cortex and posterior cingulate cortex extending into the precuneus, codes the affective and hedonic valuation of self- and other-related emotion and pleasure (Arioli et al. 2021b; Buckner et al. 2008; Raichle and Snyder 2007; Rolls 2019; Rolls et al. 2022). The salience and default mode networks have been implicated within a plethora of negative aesthetic evaluations, including dislike, sadness, sorrow and ugliness (Kross et al. 2007; Ishizu and Zeki 2011, 2017; Osaka et al. 2012; Vessel et al. 2012; Flexas et al. 2014; Vartanian et al. 2015). Concerning the behavioral motivation system (Nadal and Skov 2025), aesthetically evaluating an artwork as ugly revealed the recruitment of motor-related regions (Kawabata and Zeki 2004; Di Dio et al. 2007, 2011), perhaps reflecting bodily engagement towards experiencing an aversive affective tone of negative aesthetic evaluations (Kawabata and Zeki 2004). If more research was available within the field, the NAE meta-analysis may had further revealed clusters situated within the sensory valuation of displeasure towards visual artwork.

Of interest, Ishizu and Zeki (2011) suggested that the medial orbitofrontal cortex (mOFC) may hedonically code pleasure within a non-linear fashion, inducing a similar functionality towards both negative and positive versus neutral aesthetic evaluations. Prior research has shown that patterns in occipital activity in conjunction with the mOFC are reflected within judging a painting’s beauty (Feng et al. 2021a; Ishizu and Zeki 2011; Munar et al. 2012) yet also ugliness (Rasche et al. 2023; see also Mansueto et al. 2025 for non-artistic stimuli). Likewise, viewing images of artwork versus photographic replications lead to a higher recruitment of the ventral striatum, which was driven by patterns of occipital activity (Lacey et al. 2011). As evident by the extensive past literature assessing positive aesthetic experience (Brown et al. 2011; Vartanian and Skov 2014; Boccia et al. 2016; Chuan-Peng et al. 2020; Feng et al. 2021a; Sacheli et al. 2022; Lou et al. 2025; Vartanian et al. 2025), domain general systems have been extensively implicated within aesthetic engagement, yet their role in negative aesthetic evaluations needs further research.

Therefore, the assignment of hedonic value, of either displeasure or pleasure, towards visual artwork may recruit similar neural systems, yet the differences in negative and positive aesthetic evaluations may rely on a differing employment of neural systems inherent to the type of aesthetic engagement a viewer undergoes (Pelowski et al. 2016, 2017). Nonetheless, the right fusiform gyrus deems significant for the evaluation of a visual stimulus as displeasurable and may appropriately engender the recruitment of neural systems surmising *how* an individual engages in relation to the contextual affordances of the visual stimulus (Nadal and Skov 2025). In the case of visual artwork, this classification may present a schematic basis to interact with the artwork in a distant yet aesthetic manner (Menninghaus et al. 2017) and may promote awareness that the artwork’s content stems from a secondhand experience versus a firsthand experience (Mazzocut-Mis 2021).

### Limitations

Notably, various limitations ask the reader to take the reported results with caution. Outside the aforementioned issues in power, our NAE study pool is highly heterogeneous and spans across a broad range of visual modalities, such as different painting genres, sculptures, and architecture; therefore, the results risk an overgeneralization of neural activation towards negative aesthetic evaluation that may differ as specified by each modality. Another limitation lies within the exclusion of studies investigating aesthetic evaluations of non-artistic visual stimuli (e.g. faces and landscapes). The contextual effects of artwork status (e.g. Kirk et al. 2009) and aesthetic evaluation (e.g. Ishizu and Zeki 2013) have been shown to recruit different neural systems when engaging with a visual stimulus; therefore, aesthetic task requirements may alter a viewer’s engagement across stimuli categories (see Yeh and Peng 2019 for aesthetic evaluations towards everyday objects). Nonetheless, all the studies included within the NAE contrasted negative versus positive aesthetic evaluations, thus providing an inherent control of task bias within the NAE study bin.

On the other hand, difficulty arises within selecting a set of studies that provide aesthetic evaluations towards non-artistic objects. Indeed, most studies addressing aesthetic evaluations outside visual artwork investigate facial stimuli (Chuan-Peng et al. 2020; Martin-Loches et al. 2014), thus aesthetic evaluations towards non-artistic stimuli may greatly skew neural activation towards the social brain. In turn, we comprised a homogeneous control analogous to displeasure by including studies that only assessed neural activations from negative IAPS images that are validated to induce negative affect within the viewer (Lang et al. 2005; Chang et al. 2015). Of note, both the NAE and N-IAPS had a varying degree of object categories, consisting of human and/or non-human content. This heterogeneity of social stimuli across negative visual experience may have significantly impacted our results; however, our aim was not to dissociate between classes across artistic and non-artistic stimuli yet was to provide a foundation of the neural correlates of displeasure. Nonetheless, the segregation of NAE studies based on differing types of negative aesthetic evaluations or classes of visual artwork may had been promising to inquire into the specific engagement of negative emotional networks (i.e. disgust or pain) within aesthetic experience (see Nadal and Skov 2025). Likewise, a meta-analysis investigating the neural correlates of objective (i.e. related to the depicted content) negativity in visual artwork (e.g. artwork depicting pain, see Ardizzi et al. 2021) would have been a promising contribution to the field. Unfortunately, there were not an adequate number of studies on these topics to conduct proper meta-analyses. These endeavors are left as a path for future research.

Accordingly, our meta-analysis showcases studies that were either only reported within literature or were received after contacting the authors of published studies. Significant results that were not reported or lacked a need to disclose (i.e. publication bias) may have resulted in a skewed level of activation in our findings. Indeed, positivity has dominated the field of neuroaesthetics; therefore, results and analyses that may counter such positivity may had been forfeited from publication, under-representing literature for negative aesthetic experience and possibly leading to a negative impact on the power of the VAE study pool and its associated results. However, our meta-analysis on negative aesthetic evaluations alongside meta-analyses on positive aesthetic experience (Boccia et al. 2016; Brown et al. 2011; Chuan-Peng et al. 2020; Feng et al. 2021a; Lou et al. 2025; Sacheli et al. 2022; Vartanian et al. 2025; Vartanian and Skov 2014) allows a qualitative assessment for future research exploring intricacies between the two.

## Conclusions

The negative aesthetic evaluation of visual artwork may employ similar visual perceptive processes as in experiencing negative affect from non-artistic images. Considerable research exploring the neural correlates of the positive aesthetic experience has been established, yet our lack of power in the NAE, and consequently the reported results, may lie within a general lack of interest towards the neural correlates underlying negative aesthetic evaluations towards artwork. Therefore, researchers within neuroaesthetics should be moved to expand their focus towards other aspects of aesthetic experience, such as artwork depicting or inducing negative emotion which has been shown time and time again to have a lasting effect on both viewers and artists alike.

## Data Availability

The data underlying this article will be shared on reasonable request to the corresponding author.
